# Human-relevant disease models to accelerate the development of nucleic acid-based therapies and other advanced therapeutics

**DOI:** 10.1242/dmm.053109

**Published:** 2026-10-26

**Authors:** Marguerite M. Colin, James E. D. Thaventhiran, Anne E. Willis

**Affiliations:** MRC Toxicology Unit, University of Cambridge, Tennis Court Rd, Cambridge, CB2 1QR, UK

**Keywords:** *In vitro* models, *Ex vivo* models, Organoids, Perfused organs, Nucleic acid-based therapies

## Abstract

Advanced therapeutics, including programmable nucleic acid-based therapies (NATs), such as small interfering RNAs (siRNAs), antisense oligonucleotides (ASOs), CRISPR-Cas 9 editing systems, modified *in vitro* transcribed messenger RNAs (mRNAs) and Adeno-associated viruses (AAVs), have the potential to transform healthcare. However, while there have been considerable successes, there have also been notable clinical failures in early-stage human trials, which slowed down the progression of these therapeutic platforms. Multiple trials of NATs have been halted or discontinued, or approved products carry black box warnings as unforeseen or underestimated toxicities and poor efficacy have been observed in humans. There are a variety of reasons for failure of NATs, one of which is that many preclinical studies lack disease modelling in a human-relevant manner and, therefore, do not capture disease-specific effects on therapy tolerability and efficacy. This Perspective provides a short overview of why better human disease modelling is required to understand the potential therapeutic liabilities of NATs across modalities.

## Teaser

**Summary:** Organoids and normothermic machine perfusion of human donor tissue represent compelling alternatives to animal models by emulating diseased or aged patients that nucleic acid-based therapies are designed to treat.

**Figure:**
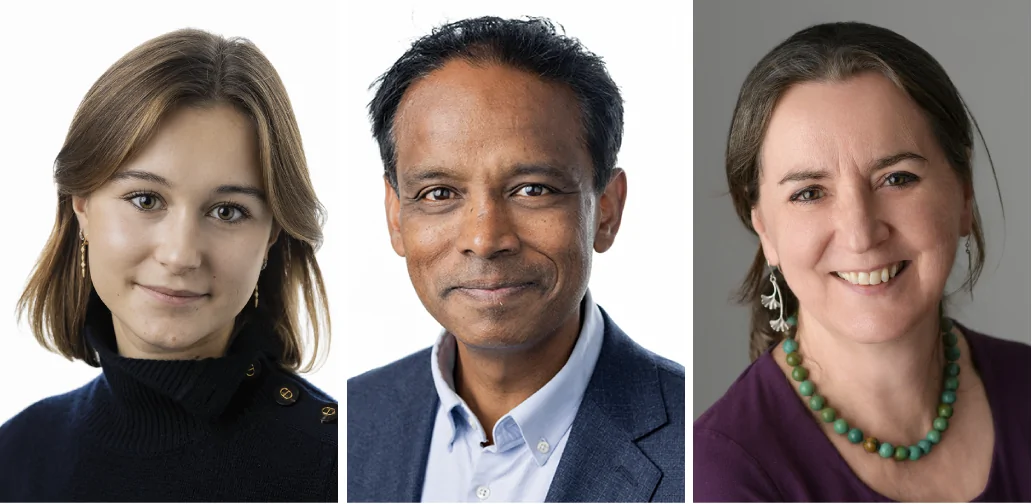
Marguerite M. Colin, James E. D. Thaventhiran and Anne E. Willis (left to right).

## INTRODUCTION

Advanced therapeutics, including nucleic acid-based therapies (NATs) and Adeno-associated viruses (AAVs), provide new treatment options for a wide range of diseases owing to their precise targeting and personalisation, and their inherent programmability, which simplifies development and manufacturing processes (Khvorova and Watts, 2017). In the last decade, there has been remarkable progress, and the U.S. Food and Drug Administration's (FDA), and the UK Medicines and Healthcare products Regulatory Agency (MHRA) have approved several of these therapies, highlighting their potential to transform human healthcare (Collotta et al., 2023; Khvorova and Watts, 2017). Yet, despite successes, there have been significant failures resulting from treatment-emergent adverse events (TEAEs) leading to withdrawal of Phase II/III clinical trials (Balwani et al., 2020; Shieh et al., 2023). The most common TEAEs of NATs in clinical trials that contribute to late-stage failures are hepato-, nephron- (renal) and immunotoxicity.

In general, failures result from an incomplete understanding of the mechanisms of toxicity, and an inability to predict the combination of chemistries and sequences of NATs that is likely to trigger adverse outcome pathways (AOPs). These issues can be addressed by carrying out mechanistic, safety and toxicology in parallel with their development, with each process informing the other. However, insufficient ability of preclinical models to predict clinical outcomes is hindering the translation of NATs, particularly in allowing their transition to treat common diseases (Chi et al., 2022; Fontana et al., 2024). A key issue is that preclinical screens are routinely conducted in healthy, young mice or non-human primates. While healthy, or even partially diseased young mice are useful for assessing pharmacodynamics, pharmacokinetics and biodistribution (Bogani et al., 2026), traditional *in vivo* preclinical models lack the essential co-founding variables that are pertinent to human health (Hansen and Hansen, 2021; Pavithra et al., 2016; Poupeau et al., 2019). It is well accepted that aging, disease and even exercise are well known to impact human health, drug bioavailability and efficacy; yet these factors are not well modelled in preclinical screens (Walter-Sack and Klotz, 1996). These variables are structurally difficult to model owing to the practical realities of preclinical housing: long-term housing can be prohibitively expensive, and controlled diet and restricted exercise space produces animals that have limited physiological resemblance to patients. Conversely, disease pathology in human tissues, such as fibrosis, can affect their exposure to drugs, therefore, potentially affecting toxicity or efficacy. For example, in experimental rodent models of liver fibrosis, reduced hepatic blood flow and expression of members of the cytochrome P450 (CYP) enzyme superfamily increased tissue exposure to the beta blocker carvedilol and the Ca^2+-^channel blocker verapamil, and reduced drug clearance (Chen et al., 2008; El-Demerdash et al., 2017). Similarly, clinical data associate high fibrosis risk or pre-existing liver disease with more severe adverse drug reactions and drug-induced liver injury (Henze et al., 2026; Ou et al., 2026).

Hence, the physiological state of controlled inbred *in vivo* models does not reflect the human state, especially in aged, diseased or comorbid patients, thereby participating to late-stage and costly failures in therapeutic development.

Furthermore, pathogen-free housing of mice removes microbiome diversity, infection history, environmental exposures to allergens or toxins and the trained immune memory, altering receptor expression, metabolic enzyme activity and epigenetic state in ways entirely invisible in controlled rodent studies (Hansen and Hansen, 2021; Pavithra et al., 2016; Poupeau et al., 2019). Hence, the physiological state of controlled inbred *in vivo* models does not reflect the human state, especially in aged, diseased or comorbid patients, thereby participating to late-stage and costly failures in therapeutic development (Chi et al., 2022; Shieh et al., 2023; Zarezadeh et al., 2021).

In particular, the limited cross-species reactivity of NATs, which affects both treatment efficacy and toxicity, further restricts translation to the human patient (Andersson, 2022), leading to many late-stage failures (Shieh et al., 2023; Van Poelgeest et al., 2015).

## Toxicity from advanced therapeutics

Unanticipated hepato-, nephron- and immunotoxicity, not predicted in preclinical models, is restricting the development of NATs and/or AAVs, leading to clinical trial failure. For example, in the ASPIRO trial for X-linked myotubular myopathy, patients died from liver injury after AAV8-mediated delivery of myotubularin 1 gene therapy (Shieh et al., 2023). Subsequently, Pannia et al. (2026) revealed that environmental factors, importantly diet, were key to the unanticipated liver disease in mutant or wild-type mice. This was characterised by altered bile acid composition, elevated levels of plasma transaminases and pathological liver injury or cholestasis. Another example is the development of mipomersen, an antisense oligonucleotide (ASO) gapmer that targets apolipoprotein B-100 mRNA, reducing low-density lipoprotein cholesterol (LDL-C) and other atherogenic lipoproteins (Santos et al., 2015; Hashemi et al., 2014). While clear and effective reduction in LDL-C was achieved in preclinical mouse models, unexpected severe elevation of liver transaminases and hepatic steatosis were observed in clinical trials at therapeutic dose (Hashemi et al., 2014; Santos et al., 2015). These toxicity signals were underestimated in rodent preclinical models because of differences in lipid metabolism and led to withdrawal of approval (Hashemi et al., 2014; Santos et al., 2015). Moreover, whereas givosiran, a small interfering RNA (siRNA) targeting hepatic 5′-aminolevulinic acid synthase 1 (*ALAS1*) has been successfully used to treat acute hepatic porphyria, elevated levels of liver-injury biomarkers and renal toxicity were reported in the metabolically diseased human organs following treatment (Balwani et al., 2020). However, liver or renal injury was not detected in the rodent clinical model with healthy organs, which prompted regulators to require post-approval pharmacovigilance, i.e. systemic monitoring of the drug safety in the patient population (Balwani et al., 2020). Another example where extensive pharmacovigilance is required is for inotersen, an ASO that targets transthyretin (*TTR*) for the treatment of polyneuropathy, that carries a black box warning because it can cause severe thrombocytopenia (Bhamra et al., 2025; Domínguez Senín et al., 2022). In the case of SPC5001, a gapmer ASO that targets proprotein convertase subtilisin/kexin type 9 (*PCSK9*), preclinical models did neither predict renal injury nor injection-site reactions, once again underestimating potential toxicity observed in clinical trials (Van Poelgeest et al., 2015).

Healthy, young and housed *in vivo* models have repeatedly failed to capture the toxicity of novel therapeutics, and improved models must be adopted to accelerate their development.

In all cases, trial failure was associated with poor physiological relevance of the preclinical models, where diet, age and environmental exposure was not representative of human pathophysiology. Healthy, young and housed *in vivo* models have repeatedly failed to capture the toxicity of novel therapeutics, and improved models must be adopted to accelerate their development (Fig. 1).

**Fig. 1.**
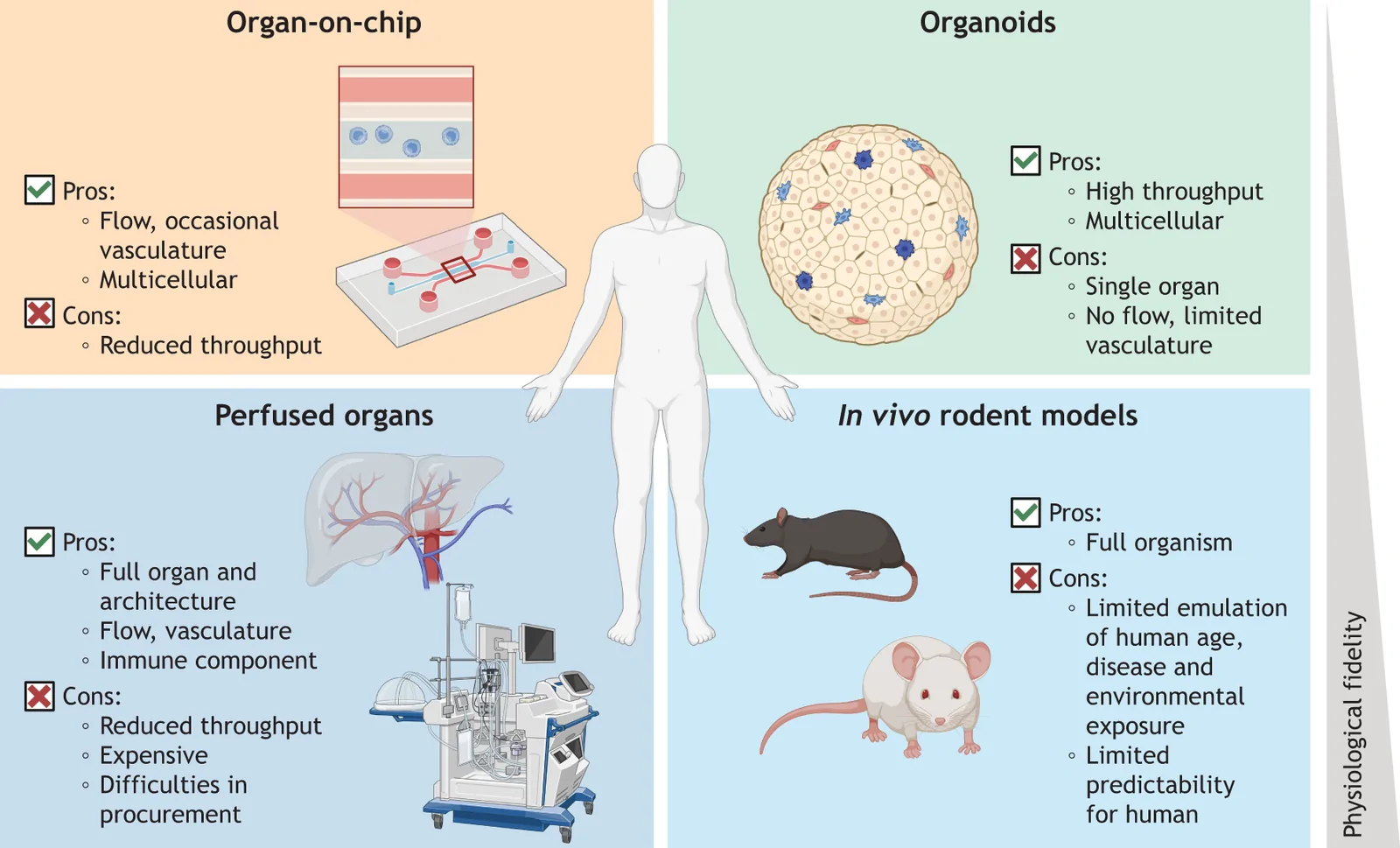
**Novel *in vitro* models are necessary to better capture human physiology and disease to accelerate novel nucleic acid-based therapies development.** Advanced therapeutics, including programmable NATs, hold tremendous potential but their clinical translation has been hindered by costly clinical failures due to unforeseen or underestimated toxicity or poor efficacy compared to *in vivo* preclinical models. New approach methodologies (NAMs), including organoids, organ-on-chip or organs that have undergone normothermic machine perfusion (NMP), can better emulate human age, disease or immune state, and environmental exposures that are critical to model for appropriate assessment of drug responses and capturing potential toxicity. While each of these models bear advantages and challenges, they represent, when used individually or in combination, a compelling and credible path towards preclinical drug-safety testing that appropriately reflects the patients being treated.

## Human-relevant disease models for safer medicines

### Patient-derived *in vitro* organoids: opportunities and challenges

Derived from human stem cells or patient-derived cells, organoids can simulate the functional as well as structural features of organs, potentially modelling some aspects of disease and age, which makes them intrinsically relevant preclinical models (Li and Izpisua Belmonte, 2019). Organoids as a drug-development screening platform are well validated, with applications for advanced therapeutics where attrition is associated with TEAEs. In structural cardiotoxicity, 3D cardiac microtissues, including endothelial cells, have enabled screening of a wider range of cardiotoxic compounds. Combining 3D cardiac microtissue culture with metabolomics allows biochemical signatures of injury to be defined, and can be used for earlier prediction of toxicity (Bowen et al., 2025). Kidneys, with tubule-on-a-chip technology, have also captured human-relevant toxicity. Nieskens et al. (2021) used chip-cultured human renal proximal tubule cells exposed to the PCSK9-targeting ASO SPC5001 and were able to recapitulate kidney injury markers observed in human trials. Furthermore, human pluripotent stem cell-derived organoids can detect hepatotoxic responses with greater sensitivity than 2D cultures and resolve inter-individual differences in a drug-induced liver injury (DILI) screen (Shinozawa et al., 2021). Incorporating patient-derived cells into such models can be advantageous when heterogenous responses need to be explored. As described by Koido and colleagues, screening of multi-donor organoids allowed the identification of toxic compounds and the potential mechanism of toxicity among donors, insights that could not be derived from healthy and young model organisms (Koido et al., 2020).

Despite these advantages, and while some studies include the vasculature or immune cells (Browne et al., 2021; Quintard et al., 2024), diseased organoids usually represent a single tissue and its individual pathology. Stem-cell-derived organoid models can also remain immature, and the costs associated with procuring aged patient cells can be prohibitive. Additionally, organoids often lack the resolution to examine crosstalk between organs and metabolites or cytokines, or the circulatory stress, both essential in understanding the true toxicity and efficacy of a therapeutic. Advances in organ-on-chip technology could allow to capture inter-tissue interactions, and 3D bioprinting could provide some solutions with regards to reproducibility, but throughput limits their applicability from large therapeutic screens (Mazzocchi et al., 2019; Schneider et al., 2021). Conversely, the ability to scale traditional organoids makes, them essential as a first line screening tool, but additional methodologies are needed to truly recapitulate the human patient.

### *Ex vivo* organs perfusion as the new preclinical frontier?

NMP of human organs represents a new frontier for assessing advanced therapeutics in a human-relevant context. NMP allows maintenance of donor organs, such as liver, kidney or gastrointestinal tract, healthy or diseased, in a metabolically active and haemodynamically supported state. Importantly, these perfused organs can emulate the physiological and systemic response to therapeutics in real time, providing human-relevant insights (Ceresa et al., 2018). In NMP organs, therapeutic delivery, function, immune reaction and clearance can be faithfully controlled and investigated over a prolonged preservation period. Moreover, these organs are harvested from donors, and vary in age, environmental exposure, immune history, comorbidity and concomitant treatment, providing the optimal testing grounds for screening to help develop safer medicines for heterogenous patient populations. For example, diseased or aged organs have been successfully deployed (Mergental et al., 2020) and have great potential for investigating drug response in a translationally relevant environment.

Several studies demonstrate the clinical relevance of the NMP liver platform (Nasralla et al., 2018; Ravikumar et al., 2016). When combined with cholangiocyte organoids, NMP-preserved livers have been shown to contribute to the repair of diseased bile ducts (Sampaziotis et al., 2021). NMP-preserved livers can, therefore, recapitulate clinically meaningful endpoints, such as bile production, lactate clearance, bile pH and ATP recovery. Importantly, NMP livers can also be used as an investigative platform for drugs and advanced therapeutics (Ceresa et al., 2018; de Vries et al., 2019). The development of long-term NMP of human split livers for translational research has further enhanced their utility, allowing paired control and titrated experiments to be performed within the same human sample (Lau et al., 2024). Moreover, it is now possible to preserve injured human livers for over a week (Eshmuminov et al., 2020) and, at these timepoints, delayed onset of toxicity or adaptive responses can be more fully assessed.

Similarly, NMP kidneys have the potential to model both healthy and diseased tissue. Ferdinand et al. (2021) demonstrated that cytokine absorption in preserved NMP kidneys delays the development of graft-function-associated inflammatory gene signatures, thereby modulating a validated clinical injury pathway in damaged human tissue. Additionally, therapeutic delivery to a damaged organ can be assessed in a setting that preserves human tissue architecture, perfusion and resident immune components, thereby outperforming traditional preclinical models that lack competent immune reaction and clearance. NMP kidneys have been used to examine the efficacy and safety of advanced therapeutics, and it has been shown that microRNA antagonist delivery alters gene expression profiles associated with ischaemic tubular damage (Thompson et al., 2022). Other examples of their use for safety testing include exposure of transplantation-declined human kidneys to the mitochondria-targeted antioxidant mitoquinone mesylate (MitoQ), allowing the pharmacological effects to be investigated at human-relevant dosage (Hamed et al., 2021).

Therefore, NMP allows for the use of controlled and pathologically advanced tissues to identify delayed toxicities, adaptative responses and pathophysiological dynamics that cannot be generally captured.

Therefore, NMP allows for the use of controlled and pathologically advanced tissues to identify delayed toxicities, adaptative responses and pathophysiological dynamics that cannot be generally captured (Hosgood et al., 2023). Importantly, while NMP requires extensive infrastructure that can be costly, there is potential for scalability. The organs used for NMP are typically deemed unsuitable for transplantation and would otherwise be discarded, with their supply far exceeding that of transplantable organs. Organs from donors with metabolic disease that have been exposed to various environmental factors, have accumulated the biological history that animal models cannot replicate. Furthermore, unlike conventional preclinical models where each condition requires a separate animal, the ability to run multiple compounds and paired controls within a single perfused organ that is unsuitable for transplant, makes NMP a scalable drug-testing platform. As the NMP technology continues to be developed, other organs, in addition to kidney, gut and liver discussed here, could be perfused (Barlow et al., 2015), opening further the horizon of possibles for NMP organs as routine testing grounds for drug development.

## Future considerations: regulatory frameworks and necessary benchmarks

With the FDA Modernisation Act 2.0 (https://www.congress.gov/bill/117th-congress/senate-bill/5002/text?s=2&r=3&hl=S.5002), and the MHRA guidance for non-animal methods [Medicines and Healthcare products Regulatory Agency (MHRA), 2025], the regulatory landscape has taken a clear stance towards potentially phasing out animal testing. The novel methodologies discussed here could provide an alternative way to move beyond traditional preclinical models, by modelling age, comorbidities and environmental exposure, variables that are inherently difficult to assess in rodent models. The use of multiple complementary model systems in drug development, rather than reliance on a single *in vivo* rodent model, is now attainable and could be adopted as a new standard. Capturing these human-relevant parameters is essential in enhancing advanced therapeutic development and selecting the right candidate for clinical trials. Nevertheless, a defined set of benchmarks and standards is necessary to further push adoption of these models. Standardised organoid production and, more specifically, disease-state characterisation need to be implemented to permit generation of reproducible and robust data that, in turn, can be used for regulatory approval. For perfused organs, a toxicity biomarker index for conditions of interest needs to be defined and widely adopted across academia and industry, and accepted by regulators. Moving away from the traditional healthy and young *in vivo* models will require coordination among all stakeholders, and this Perspective article aimed to outline the opportunities offered by novel *in vitro* and *ex vivo* methods.

## Conclusion

Failures of conventional preclinical models to predict clinical outcomes underscore the urgent need for human-relevant alternatives. Organoids and normothermic machine perfusion of donor tissue can recapitulate the disease state, age and environmental exposure that define real patient populations, and offer a credible pipeline for the safety assessment of NATs. In the near term, complementary use of these novel platforms represents a pragmatic path forward, while outstanding limitations are being continuously addressed. Realising their potential will require regulators, funders and industry to come together, in order to establish validated standards and guidelines that allow data of human disease models to support clinical dossiers. The biological and engineering foundations are in place, and the priority should be to advance conversations with regulators for their wider use and adoption.

## References

[DMM053109C1] Andersson, P. (2022). Preclinical safety assessment of therapeutic oligonucleotides. In: *Antisense RNA Design, Delivery, and Analysis* (*Methods in Molecular Biology)*, (ed. V. Arechavala-Gomeza and A. Garanto), pp. 355-370. Humana Press Inc. 10.1007/978-1-0716-2010-6_25 PMC9703267 35213031

[DMM053109C2] Balwani, M., Sardh, E., Ventura, P., Peiró, P. A., Rees, D. C., Stölzel, U., Bissell, D. M., Bonkovsky, H. L., Windyga, J., Anderson, K. E. et al. (2020). Phase 3 trial of RNAi therapeutic givosiran for acute intermittent porphyria. *N. Engl. J. Med.* 382, 2289-2301. 10.1056/NEJMoa1913147 32521132

[DMM053109C3] Barlow, A. D., Hamed, M. O., Mallon, D. H., Brais, R. J., Gribble, F. M., Scott, M. A., Howat, W. J., Bradley, J. A., Bolton, E. M., Pettigrew, G. J. et al. (2015). Use of ex vivo normothermic perfusion for quality assessment of discarded human donor pancreases. *Am. J. Transplant.* 15, 2475-2482. 10.1111/ajt.13303 PMC7212093 25989187

[DMM053109C4] Bhamra, J., Krishna, M., Samaan, G., Pattanayak, S. and Mukhopadhyay, S. (2025). Toxicity of antisense oligonucleotides is determined by the synergistic interplay of chemical modifications and nucleotide sequences, not by either factor alone. *Chembiochem* 26, e202500584. 10.1002/cbic.202500584 41078095

[DMM053109C5] Bogani, D., Hooper, K. and Sansom, O. (2026). Why in vivo models of disease remain indispensable. *Dis. Model. Mech.* 19, dmm052997. 10.1242/dmm.052997 PMC13225216 42084366

[DMM053109C6] Bowen, T. J., Hall, A. R., Lloyd, G. R., Smith, M. J., Weber, R. J. M., Wilson, A., Pointon, A. and Viant, M. R. (2025). Discovering a predictive metabolic signature of drug-induced structural cardiotoxicity in cardiac microtissues. *Arch. Toxicol.* 99, 3379-3392. 10.1007/s00204-025-04074-4 PMC12367874 40379885

[DMM053109C7] Browne, S., Gill, E. L., Schultheiss, P., Goswami, I. and Healy, K. E. (2021). Stem cell-based vascularization of microphysiological systems. *Stem Cell Rep.* 16, 2058-2075. 10.1016/j.stemcr.2021.03.015 PMC8452487 33836144

[DMM053109C8] Ceresa, C. D. L., Nasralla, D. and Jassem, W. (2018). Normothermic machine preservation of the liver: state of the art. *Curr. Transplant. Rep.* 5, 104-110. 10.1007/s40472-018-0186-9 PMC5843699 29564207

[DMM053109C9] Chen, M., Xu, D., Hu, X. L. and Wang, H. (2008). Effects of liver fibrosis on verapamil pharmacokinetics in rats. *Clin. Exp. Pharmacol. Physiol.* 35, 287-294. 10.1111/j.1440-1681.2007.04826.x 17973928

[DMM053109C10] Chi, L. H., Burrows, A. D. and Anderson, R. L. (2022). Can preclinical drug development help to predict adverse events in clinical trials? *Drug Discov. Today* 27, 257-268. 10.1016/j.drudis.2021.08.010 34469805

[DMM053109C11] Collotta, D., Bertocchi, I., Chiapello, E. and Collino, M. (2023). Antisense oligonucleotides: a novel Frontier in pharmacological strategy. *Front. Pharmacol.* 14, 1304342. 10.3389/fphar.2023.1304342 PMC10690781 38044945

[DMM053109C12] de Vries, Y., Matton, A. P. M., Nijsten, M. W. N., Werner, M. J. M., van den Berg, A. P., de Boer, M. T., Buis, C. I., Fujiyoshi, M., de Kleine, R. H. J., van Leeuwen, O. B. et al. (2019). Pretransplant sequential hypo- and normothermic machine perfusion of suboptimal livers donated after circulatory death using a hemoglobin-based oxygen carrier perfusion solution. *Am. J. Transplant.* 19, 1202-1211. 10.1111/ajt.15228 PMC6590255 30588774

[DMM053109C13] Domínguez Senín, L., Borrachero Garro, C., Sánchez Gómez, E. and Santos-Rubio, M. D. (2022). Inotersen and severe thrombocytopenia: Two case reports and review. *Int. J. Clin. Pharmacol. Ther.* 60, 311-316. 10.5414/CP204190 35343431

[DMM053109C14] El-Demerdash, E., Abdel-Sattar, S. A., El-Bakly, W. M. and Mohamed, E. A. (2017). Antifibrotic effects of carvedilol and impact of liver fibrosis on carvedilol pharmacokinetics in a rat model. *Eur. J. Drug Metab. Pharmacokinet.* 42, 767-779. 10.1007/s13318-016-0391-9 28012025

[DMM053109C15] Eshmuminov, D., Becker, D., Bautista Borrego, L., Hefti, M., Schuler, M. J., Hagedorn, C., Muller, X., Mueller, M., Onder, C., Graf, R. et al. (2020). An integrated perfusion machine preserves injured human livers for 1 week. *Nat. Biotechnol.* 38, 189-198. 10.1038/s41587-019-0374-x PMC7008032 31932726

[DMM053109C16] Ferdinand, J. R., Hosgood, S. A., Moore, T., Ferro, A., Ward, C. J., Castro-Dopico, T., Nicholson, M. L. and Clatworthy, M. R. (2021). Cytokine absorption during human kidney perfusion reduces delayed graft function–associated inflammatory gene signature. *Am. J. Transplant.* 21, 2188-2199. 10.1111/ajt.16371 PMC8246774 33098231

[DMM053109C17] Fontana, M., Solomon, S. D., Kachadourian, J., Walsh, L., Rocha, R., Lebwohl, D., Smith, D., Täubel, J., Gane, E. J., Pilebro, B. et al. (2024). CRISPR-Cas9 gene editing with nexiguran ziclumeran for ATTR cardiomyopathy. *N. Engl. J. Med.* 391, 2231-2241. 10.1056/NEJMoa2412309 39555828

[DMM053109C18] Hamed, M., Logan, A., Gruszczyk, A. V., Beach, T. E., James, A. M., Dare, A. J., Barlow, A., Martin, J., Georgakopoulos, N., Gane, A. M. et al. (2021). Mitochondria-targeted antioxidant MitoQ ameliorates ischaemia-reperfusion injury in kidney transplantation models. *Br. J. Surg.* 108, 1072-1081. 10.1093/bjs/znab108 33963377

[DMM053109C19] Hansen, A. K. and Hansen, C. H. F. (2021). The microbiome and rodent models of immune mediated diseases. *Mamm. Genome* 32, 251-262. 10.1007/s00335-021-09866-4 PMC8012743 33792799

[DMM053109C20] Hashemi, N., Odze, R. D., McGowan, M. P., Santos, R. D., Stroes, E. S. G. and Cohen, D. E. (2014). Liver histology during Mipomersen therapy for severe hypercholesterolemia. *J. Clin. Lipidol.* 8, 606-611. 10.1016/j.jacl.2014.08.002 PMC4268487 25499943

[DMM053109C21] Henze, J., Dormann, H., Schneider, C. V., Meeßen, C., Knüppel-Ruppert, A., Kriegisch-Stumpf, A., Schwab, M., Stingl, J. C. and Just, K. S. (2026). Association of high risk of liver fibrosis in patients with serious outcomes of adverse drug reactions. *Eur. J. Clin. Pharmacol.* 82, 128. 10.1007/s00228-026-04057-z PMC13102728 42020558

[DMM053109C22] Hosgood, S. A., Callaghan, C. J., Wilson, C. H., Smith, L., Mullings, J., Mehew, J., Oniscu, G. C., Phillips, B. L., Bates, L. and Nicholson, M. L. (2023). Normothermic machine perfusion versus static cold storage in donation after circulatory death kidney transplantation: a randomized controlled trial. *Nat. Med.* 29, 1511-1519. 10.1038/s41591-023-02376-7 PMC10287561 37231075

[DMM053109C23] Khvorova, A. and Watts, J. K. (2017). The chemical evolution of oligonucleotide therapies of clinical utility. *Nat. Biotechnol.* 35, 238-248. 10.1038/nbt.3765 PMC5517098 28244990

[DMM053109C24] Koido, M., Kawakami, E., Fukumura, J., Noguchi, Y., Ohori, M., Nio, Y., Nicoletti, P., Aithal, G. P., Daly, A. K., Watkins, P. B. et al. (2020). Polygenic architecture informs potential vulnerability to drug-induced liver injury. *Nat. Med.* 26, 1541-1548. 10.1038/s41591-020-1023-0 PMC8482794 32895570

[DMM053109C25] Lau, N. S., McCaughan, G., Ly, M., Liu, K., Crawford, M. and Pulitano, C. (2024). Long-term machine perfusion of human split livers: a new model for regenerative and translational research. *Nat. Commun.* 15, 9809. 10.1038/s41467-024-54024-4 PMC11557707 39532864

[DMM053109C26] Li, M. and Izpisua Belmonte, J. C. (2019). Organoids — Preclinical models of human disease. *N. Engl. J. Med.* 380, 569-579. 10.1056/NEJMra1806175 30726695

[DMM053109C27] Mazzocchi, A., Soker, S. and Skardal, A. (2019). 3D bioprinting for high-throughput screening: drug screening, disease modeling, and precision medicine applications. *Appl. Phys. Rev.* 6, 011302. 10.1063/1.5056188 PMC7968875 33738018

[DMM053109C28] Mergental, H., Laing, R. W., Kirkham, A. J., Perera, M. T. P. R., Boteon, Y. L., Attard, J., Barton, D., Curbishley, S., Wilkhu, M., Neil, D. A. H. et al. (2020). Transplantation of discarded livers following viability testing with normothermic machine perfusion. *Nat. Commun.* 11, 2939. 10.1038/s41467-020-16251-3 PMC7298000 32546694

[DMM053109C29] Nasralla, D., Coussios, C. C., Mergental, H., Akhtar, M. Z., Butler, A. J., Ceresa, C. D. L., Chiocchia, V., Dutton, S. J., García-Valdecasas, J. C., Heaton, N. et al. (2018). A randomized trial of normothermic preservation in liver transplantation. *Nature* 557, 50-56. 10.1038/s41586-018-0047-9 29670285

[DMM053109C30] Nieskens, T. T. G., Magnusson, O., Andersson, P., Söderberg, M., Persson, M. and Sjögren, A. K. (2021). Nephrotoxic antisense oligonucleotide SPC5001 induces kidney injury biomarkers in a proximal tubule-on-a-chip. *Arch. Toxicol.* 95, 2123-2136. 10.1007/s00204-021-03062-8 33961089

[DMM053109C31] Ou, X., Wu, Y., Cheng, Q., Yin, W., Rong, C., He, S., Wei, L. and Yu, P. (2026). Clinical profiles and risk predictors of drug-induced liver injury from pirfenidone and nintedanib used to treat idiopathic pulmonary fibrosis: a real-world analysis of a tertiary Chinese hospital cohort combined with FAERS data mining. *Transl. Gastroenterol. Hepatol.* 11, 9. 10.21037/tgh-25-104 PMC12887313 41675318

[DMM053109C32] Pannia, E., Simonian, R., Sabha, N., Maani, N., Zhu, Y., Karolczak, S., Raileanu, V., Bottiglieri, T., Andreoletti, G., Edgar, R. D. et al. (2026). AAV8 gene therapy and dietary insults together precipitate cholestatic liver disease in a mouse model of X-linked myotubular myopathy. *Sci. Transl. Med.* 18, 1483. 10.1126/scitranslmed.adr1483 41706871

[DMM053109C33] Pavithra, F., Cheng, S. L., Klaassen, C. D. and Cui, J. Y. (2016). Regulation of hepatic drug-metabolizing enzymes in germ-free mice by conventionalization and probiotics. *Drug Metab. Dispos.* 44, 262-274. 10.1124/dmd.115.067504 PMC4746487 26586378

[DMM053109C34] Poupeau, A., Garde, C., Sulek, K., Citirikkaya, K., Treebak, J. T., Arumugam, M., Simar, D., Olofsson, L. E., Bäckhed, F. and Barrès, R. (2019). Genes controlling the activation of natural killer lymphocytes are epigenetically remodeled in intestinal cells from germ-free mice. *FASEB J.* 33, 2719-2731. 10.1096/fj.201800787R PMC6338647 30303739

[DMM053109C35] Quintard, C., Tubbs, E., Jonsson, G., Jiao, J., Wang, J., Werschler, N., Laporte, C., Pitaval, A., Bah, T. S., Pomeranz, G. et al. (2024). A microfluidic platform integrating functional vascularized organoids-on-chip. *Nat. Commun.* 15, 1452. 10.1038/s41467-024-45710-4 PMC10873332 38365780

[DMM053109C36] Ravikumar, R., Jassem, W., Mergental, H., Heaton, N., Mirza, D., Perera, M. T. P. R., Quaglia, A., Holroyd, D., Vogel, T., Coussios, C. C. et al. (2016). Liver transplantation after ex vivo normothermic machine preservation: a phase 1 (First-in-Man) clinical trial. *Am. J. Transplant.* 16, 1779-1787. 10.1111/ajt.13708 26752191

[DMM053109C37] Sampaziotis, F., Muraro, D., Tysoe, O. C., Sawiak, S., Beach, T. E., Godfrey, E. M., Upponi, S. S., Brevini, T., Wesley, B. T., Garcia-Bernardo, J. et al. (2021). Cholangiocyte organoids can repair bile ducts after transplantation in the human liver. *Science (1979)* 371, 839-846. 10.1126/science.aaz6964 PMC7610478 33602855

[DMM053109C38] Santos, R. D., Duell, P. B., East, C., Guyton, J. R., Moriarty, P. M., Chin, W. and Mittleman, R. S. (2015). Long-term efficacy and safety of mipomersen in patients with familial hypercholesterolaemia: 2-year interim results of an open-label extension. *Eur. Heart J.* 36, 566-575. 10.1093/eurheartj/eht549 PMC4344956 24366918

[DMM053109C39] Schneider, M. R., Oelgeschlaeger, M., Burgdorf, T., van Meer, P., Theunissen, P., Kienhuis, A. S., Piersma, A. H. and Vandebriel, R. J. (2021). Applicability of organ-on-chip systems in toxicology and pharmacology. *Crit. Rev. Toxicol.* 51, 540-554. 10.1080/10408444.2021.1953439 34463591

[DMM053109C40] Shieh, P. B., Kuntz, N. L., Dowling, J. J., Müller-Felber, W., Bönnemann, C. G., Seferian, A. M., Servais, L., Smith, B. K., Muntoni, F., Blaschek, A. et al. (2023). Safety and efficacy of gene replacement therapy for X-linked myotubular myopathy (ASPIRO): a multinational, open-label, dose-escalation trial. *Lancet Neurol.* 22, 1125-1139. 10.1016/S1474-4422(23)00313-7 37977713

[DMM053109C41] Shinozawa, T., Kimura, M., Cai, Y., Saiki, N., Yoneyama, Y., Ouchi, R., Koike, H., Maezawa, M., Zhang, R. R., Dunn, A. et al. (2021). High-fidelity drug-induced liver injury screen using human pluripotent stem cell–derived organoids. *Gastroenterology* 160, 831-846.e10. 10.1053/j.gastro.2020.10.002 PMC7878295 33039464

[DMM053109C42] Thompson, E. R., Sewpaul, A., Figuereido, R., Bates, L., Tingle, S. J., Ferdinand, J. R., Situmorang, G. R., Ladak, S. S., Connelly, C. M., Hosgood, S. A. et al. (2022). MicroRNA antagonist therapy during normothermic machine perfusion of donor kidneys. *Am. J. Transplant.* 22, 1088-1100. 10.1111/ajt.16929 34932895

[DMM053109C43] Van Poelgeest, E. P., Hodges, M. R., Moerland, M., Tessier, Y., Levin, A. A., Persson, R., Lindholm, M. W., Dumong Erichsen, K., Ørum, H., Cohen, A. F. et al. (2015). Antisense-mediated reduction of proprotein convertase subtilisin/kexin type 9 (PCSK9): A first-in-human randomized, placebo-controlled trial. *Br. J. Clin. Pharmacol.* 80, 1350-1361. 10.1111/bcp.12738 PMC4693495 26261033

[DMM053109C44] Walter-Sack, I. and Klotz, U. (1996). Influence of diet and nutritional status on drug metabolism. *Clin. Pharmacokinet.* 31, 47-64. 10.2165/00003088-199631010-00004 8827399

[DMM053109C45] Zarezadeh, M., Saedisomeolia, A., Shekarabi, M., Khorshidi, M., Emami, M. R. and Müller, D. J. (2021). The effect of obesity, macronutrients, fasting and nutritional status on drug-metabolizing cytochrome P450s: a systematic review of current evidence on human studies. *Eur. J. Nutr.* 60, 2905-2921. 10.1007/s00394-020-02421-y 33141242

